# Brand Equity, Tourist Satisfaction and Travel Intentions in a UNESCO Creative City of Gastronomy: A Case Study of Yangzhou, China

**DOI:** 10.3390/foods12142690

**Published:** 2023-07-13

**Authors:** Fei Jiang, Rong Huang, Qian Chen, Jinhua Zhang

**Affiliations:** 1 School of Tourism and Cuisine, Yangzhou University, Yangzhou 225127, China; jaaafei0908@163.com; 2 Plymouth Business School, University of Plymouth, Plymouth PL4 8AA, UK; rong.huang@plymouth.ac.uk (R.H.); jinhua.zhang@plymouth.ac.uk (J.Z.)

**Keywords:** brand equity, tourist satisfaction, travel intentions

## Abstract

Food is an indispensable part of destination tourism resources and attractions, playing a vital role in the marketing and promotion of the destination. Food can also be viewed as an important brand that the destination can develop. Yangzhou has been listed as a UNESCO Creative City of Gastronomy since 2019, and the government aims to enhance the food brand for the city. This study attempts to assess the impact of a destination’s food brand equity on tourist satisfaction and travel intentions, and to evaluate the potential of developing food tourism. Questionnaires were conducted with 481 tourists, followed by semi-structured interviews with 29 tourists. A structural equation modelling analysis addressed the positive relationships among destination food brand equity, tourist satisfaction and travel intentions. A qualitative analysis contributes to further clarifying the relationships of the model. Implications for theory, research and practice are discussed.

## 1. Introduction

As a medium for tourists to learn about the local culture, and an important part of the tourist experience, food is inextricably linked to tourism [1,2,3]. Nowadays, more and more tourists are motivated to travel by food, which influences their choice of destination [4,5,6]. As one of the tourist attractions of a destination, food is the material basis for tourism activities, offering novel experiences and opportunities for enjoyment during a visit [7,8]. In addition, food is also a tourism product that can enhance tourists’ sense of experience of the destination, playing an important role in influencing tourist satisfaction [9,10]. Local cuisine influences tourists’ choice of destination and plays an important role in creating destination uniqueness [11]. From the perspective of tourism development, local cuisine has great potential to improve the competitiveness of the destination [12,13]. Food tourism is now an integral part of the destination marketing strategy [13]. In order to develop tourism and contribute to the economic prosperity of the region, destinations are developing differentiated products and building competitive advantages to attract tourists [12].

Existing research shows that building a destination brand has become a key to attracting domestic and international tourists [14,15]. Research on brand management in the tourism and hospitality literature is identified as a central theme in the tourism marketing sector of the 21st century [16]. With particular concern to the tourism sector, it is vital for managers to choose the right marketing strategy that will effectively enhance the brand equity of the destination [17]. In contrast to other destination brand equity, food brand equity not only influences tourist attitudes and future behaviours [18], but also leads to a memorable post-trip experience [19]. Therefore, understanding how food brand equity influences tourist satisfaction and travel intentions becomes increasingly important.

In response to the urgency of cities as a necessary collaborative platform for promoting cultural diversity and sustainable urban development, the UNESCO Creative Cities Network was established in 2004 [20]. The network covers seven fields, including crafts and folk arts, design, film, gastronomy, literature, media arts and music. In particular, the title of Creative Cities in Gastronomy has been awarded to 49 cities worldwide, including five Chinese cities (i.e., Chengdu, Macau, Shunde, Yangzhou and Huaian) on the list for their excellence in food innovation and creativity. Due to the long history, China’s food culture has evolved into distinctive local cuisines and corresponding eating habits, leading to a diversity of food cultures in general [21]. But from a local perspective, different local cuisines have their own uniqueness [22,23], so every Chinese city has the basis and potential to build a food brand [21]. In addition, Yangzhou, as the main birthplace of Huaiyang cuisine, one of the four major cuisines, has rich gastronomic resources and food culture. Yangzhou was named a UNESCO Creative City of Gastronomy on 31 October 2019 [24]. Since receiving the title, Yangzhou has been working to enhance its food branding as a UNESCO Creative City of Gastronomy, develop its cultural and tourism industry and spread the idea of a creative city to provide new ideas for urban regeneration and development. Because of the unique flavours and exquisite skills, the famous Yangzhou cuisine has been favoured by countless diners. Therefore, Yangzhou cuisine enjoys more advantages and representativeness in establishing its food brand equity. However, with the impact of globalization, different kinds of cuisines are flooding into tourist destinations. Local cuisines have been impacted and homogenization has become more prevalent [25]. There is therefore a greater need to construct a unified and appropriate destination food brand [26], as well as to further measure whether food tourism enjoys sufficient development value for the destination.

However, few studies have focused on destination food brand equity from a tourist perspective; the dimensions of food brand equity and the relationships between food brand equity, tourist satisfaction and travel intentions remain to be explored. In addition, no previous research has paid attention to the context of a UNESCO Creative City of Gastronomy, and whether a UNESCO Creative City of Gastronomy could build a food brand to accelerate the development of the tourism industry is yet to be researched. It is crucial for tourism destinations to examine the role of food brand equity in influencing tourist satisfaction and travel intentions. Therefore, this study employed a mixed-method sequential explanatory approach, attempts to assess destination food brand equity from tourists’ perspective and an examination of its relationship with tourist satisfaction and travel intentions. The purpose was to examine the real development potential of food tourism destinations, thereby assisting in the establishment of unique food brands for the destinations.

This study has both theoretical and practical contributions. First, this study expands the application of brand equity theory by analysing the dimensions of food brand equity based on tourists’ perspective, using Yangzhou, a UNESCO Creative City of Gastronomy, as a case study, which contributes to understanding the brand equity of the UNESCO Creative City of Gastronomy. Second, this study contributes to the tourism literature by providing a model to assess the relationship between brand equity dimensions, tourist satisfaction and travel intention. Third, this study employed a mixed-method sequential explanatory approach using qualitative data to support quantitative data and provide a more comprehensive assessment of the theoretical model. Finally, this study can provide guidance for the development of Yangzhou’s food brand and management insights for other UNESCO Creative Cities of Gastronomy.

Based on the above, this study is organized as follows. It first critically reviews the existing literature related to brand equity, as well as the relationships between food brand equity, tourist satisfaction and travel intentions. Next, the methodology adopted for this study is discussed following the results and discussions. Then, conclusions and implications of the research findings are presented. Last, this study discusses the limitations and suggestions for future research.

## 2. Theoretical Background and Hypotheses’ Development

### 2.1. Brand Equity

Aaker considers brand equity as “a collection of brand assets and liabilities” related to consumers’ perceptions of the brand name, symbols or values offered by a product or service [27] (p. 15). Keller defines brand equity based on the consumer perspective as the differentiated reflection of marketing activities resulting from consumers’ knowledge of a brand that enables them to differentiate a particular brand’s product from others of the same type [28]. Brands add intangible and emotional value to a product or service, which influences consumers’ perception of the brand message and therefore their decisions. Consumers are more receptive to brand extensions when the brand has positive equity [29]. In other words, brand value is a sign that influences not only consumers’ perception of value, but also their behaviour towards their expectations by conveying value, perceived risk and familiarity with the brand image [30].

Several scholars have identified the dimensions of brand equity. Aaker classifies the dimensions of brand equity as brand image, perceived quality, brand loyalty and brand awareness to increase customer satisfaction, which is beneficial to the company’s growth [27]. Oh and Hsu proposed that brand value has several dimensions, including brand awareness, brand loyalty, brand image, perceived quality, utilitarian value, management trust, brand choice intention and brand reliability [31]. Horng et al. studied the relationships among the sub-dimensions of brand equity chosen by foreign tourists, such as brand loyalty, brand image, perceived quality and brand awareness [19]. Liu investigated the interrelationships between brand assets, and suggested that brand image mediates between brand awareness and utilitarian value [17]. These qualitative and quantitative studies have focused on or extended different aspects of Aaker’s concept [32], and his theory has been widely applied to research in a variety of fields such as hotels [33,34], tourism [35,36], destinations [37,38] and retail [39,40].

Brand equity is considered to be an important factor in creating a competitive advantage in the market and differentiating marketing strategies [34,37,41]. As the importance of brand equity increases, its field of application extends from marketing strategies to travel intentions for tourism and hospitality destinations. Several scholars have identified brand equity significance for tourism destination management [36,38,42]. It remains to be explored whether culinary brand equity generates positive marketing effects in the context of a UNESCO Creative City of Gastronomy, and how to determine the scale of the brand equity.

Food is one of the research objects in the field of tourism and destination studies. The uniqueness of Yangzhou’s food enables tourists to distinguish Yangzhou from other tourist cities. According to a survey conducted by the Yangzhou Municipal Government, 43.64% of the interviewees come to Yangzhou for the main purpose of traveling to taste food [43]. After Yangzhou was awarded the title of UNESCO Creative City of Gastronomy, 63.64% of the interviewees increased their willingness to travel to Yangzhou, and 29.09% generated their willingness to travel to Yangzhou [43].

The recognition of UNESCO has had a significant positive impact on the World Heritage Site, and provided direction for building and developing its brand [44,45]. The Creative Cities Network project a city’s cultural assets onto a global stage. There is a trend within the network for cities to use membership of the UNESCO Creative Cities Network as a quality label for themselves, to attract financial flows and to improve their position in global city rankings [46]. The UNESCO Creative City’s designation enhances a city’s soft power, builds its image and attracts domestic and international resources [47]. In the process, those cities named as Creative Cities of Gastronomy for food have a unique advantage to grow in the tourism industry by creating food brands. However, the challenges to be managed in a destination’s food brand involve the absence of centralized control over products and services delivered by numerous restaurants to individual tourists. The proactive coordination through a government has been reported to provide benefits [48]. Research has shown that becoming a UNESCO Creative City of Gastronomy helps to promote the culture of a destination through food, enhances the image of the destination and ultimately creates economic, cultural and social value for the city [49]. Yılmaz et al. showed that joining the UNESCO Creative Cities Network increased the reputation and visibility of gastronomic cities [50]. Pearson and Pearson noted an increase in the number of gastronomic tourists in cities that were awarded as a UNESCO Creative City of Gastronomy [49]. It is therefore of significant value to explore what impact food brand equity of a UNESCO Creative City of Gastronomy has had on tourists. This study attempts to evaluate the specific value of food as a destination brand asset and its role in relation to visitor satisfaction and travel intentions by adopting Aaker’s dimensional division of brand equity [27].

#### 2.1.1. Brand Image

Brand image refers to the perception of a brand in the minds of consumers, which is regarded as the rational or emotional perception of a specific brand by consumers [51]. Lassar et al. suggested that brand image is more popular than any other social aspect [52]. Brand image has also been identified as an important source and a key aspect of brand equity [28,33,38]. Cai argues that brand image building is an important component of the destination brand model [53]. Tourists create an association that combines brand image with perception, which creates a specific brand image [54]. Gartner states that the more familiar consumers are with a brand, the higher the brand equity will be [55]. At the same time, the more unique and favourable images consumers have in their memory, the stronger their association will be with that brand. Tourism destinations make extensive use of imagery in promotional materials to raise awareness of the characteristics that distinguish them from their competitors. This study speculates that the food brand image constructed by a UNESCO Creative City of Gastronomy may become an important component of food brand equity. Consequently, the first hypothesis was proposed:

**H1:**  *Brand image positively influences the brand equity of a UNESCO Creative City of Gastronomy.*

#### 2.1.2. Perceived Quality

Perceived quality is one of the important dimensions and an important attribute of brand equity [28,32,52]. Lewis and Chambers define perceived quality as a visitor’s judgement that is derived from a comparison between the visitor’s expectations and perceptions of service performance [56]. Tourism researchers often use perceived quality as a way to conceptualize brand equity [37,57,58]. According to Huang and Huang, destination brand perceived quality, the subjective judgement and satisfaction level of tourists towards the brand, is a comparison between the quality tourists expect from a destination before they arrive and the actual perceived quality developed after a series of travel experiences once they arrive, and is a subjective judgement and satisfaction level of tourists towards the brand [59]. Low and Lamb state that a high-quality brand can promote consumer purchases and influence tourists’ preferences [60]. Konecnik and Gartner identify brand quality as the main dimension of tourist-based brand equity in tourism destinations [38]. In addition, studies have also shown a positive relationship between perceived quality and tourist travel intentions [61]. In a UNESCO Creative City of Gastronomy, the ability of food consumption and food tourism experiences to meet visitor expectations is a measure of perceived quality, as well as a key component of a destination’s culinary brand equity. Accordingly, the following hypothesis was formulated:

**H2:**  *Perceived quality positively influences the brand equity of a UNESCO Creative City of Gastronomy.*

#### 2.1.3. Brand Awareness

Brand awareness is a fundamental component of brand equity [27,35,51], which reflects the prominence of a brand in the minds of tourists [32]. Recalling and understanding the brand have a significant impact on tourists’ choices [62]. Destination marketing aims to increase tourists’ awareness of a destination through advertising and the creation of a unique brand [63]. Aaker states that brand awareness is an ability that tourists identify a brand, which can have an impact on their decision making [27]. The higher the brand awareness is, the more dominant it will be in the market [64]. In addition, Xu and Mo found that tourism destination brand awareness has a significant impact on destination brand equity [65]. In addition, brand awareness is considered to be the first step in building brand equity and it further contributes to increase brand equity over time [66,67]. Increasing brand awareness among visitors to a UNESCO Creative City of Gastronomy will help increase the destination’s gastronomic brand equity. Brand awareness is an important component of food brand equity. Therefore, an hypothesis was formulated as follows:

**H3:**  *Brand awareness positively influences the brand equity of a UNESCO Creative City of Gastronomy.*

#### 2.1.4. Brand Loyalty

Brand loyalty, a core component of brand equity, is defined as the attachment of tourists to a brand [27]. Lassar et al. state that brand equity arises from consumers’ trust in brands over their competitors [52]. This trust translates into consumer loyalty and their willingness to pay a premium for the brand. The concept of loyalty is widely used in marketing strategies to measure consumers’ willingness to make repeat purchases or referrals [68]. However, the study of destination brand loyalty in the tourism, hospitality and leisure industries only began to be mentioned a decade ago. Destination brand loyalty has become an important topic for scholars to explore its relationship with perceived value, brand quality and consumer engagement [37,69]. The main goal of destination brand management is to build tourists’ loyalty. Brand loyalty benefits travel destinations in that tourists may revisit or recommend the destination to other potential tourists, such as friends or family [70]. Thus, brand loyalty, a core component of brand equity, measures the extent to which visitors are emotionally attached to the brand of a UNESCO Creative City of Gastronomy [62]. Thus, the following hypothesis was proposed:

**H4:**  *Brand loyalty positively influences the brand equity of a UNESCO Creative City of Gastronomy.*

### 2.2. The Impact of Brand Equity on Tourist Satisfaction and Travel Intentions

Satisfaction refers to the fact that satisfaction will increase when the expectations are met, namely a product or service meets or exceeds consumers’ expectations [71]. Tourist satisfaction is closely related to brand equity [72]. Iglesias and Guillen analysed the impact of quality and price on customer satisfaction and found that customers’ perception of quality had a significant influence [73]. Keith et al. argue that tourists’ perceived quality of a brand can satisfy their own needs and generate repeat purchase behaviour [74]. At the same time, brand awareness helps to encourage tourists to buy brand products, increasing the status of the brand in their minds [32], and their ability to distinguish and identify brands [14]. When brand awareness is high, the demand for that particular brand increases [75]. Based on these studies, higher food brand equity can be obtained from the brand image, perceived quality, brand awareness and brand loyalty, which is likely to increase tourist satisfaction. Hence, the following hypothesis is presented:

**H5:**  *Brand equity of a UNESCO Creative City of Gastronomy positively influences tourist satisfaction.*

Tourists’ travel intention is the result of tourists’ perception from the tourism experience [76]. As interest in the food resources of tourist destinations increases, gastronomy plays an increasingly important role in enhancing the differentiation of tourist destinations and increasing the willingness of tourists to visit the destinations [77]. According to statistics, food has become one of the most important reasons for Yangzhou to attract foreign tourists. Therefore, it is crucial to specifically explore the impact of Yangzhou food brand equity on tourist travel intentions. Camarero et al. argue that tourist destinations aim to create a unique and effective image and identity to encourage loyalty intentions among tourists so that these tourists become loyal [36]. When a brand image is created and generally accepted in the minds of tourists, it leads to a range of positive effects, generating positive expectations of quality, reliability and trust, which are then transferred from the brand image to travel intentions [77]. Murphy et al. argue that perceived quality can have a positive impact on perceived tourism value and travel intentions [78]. Kim and Kim argue that brand awareness is an important factor in generating an important prerequisite for tourist value and contributing to brand equity [33]. Lin studied consumer travel behaviour and demonstrated that brand loyalty positively influences travel intentions [62]. In summary, there are positive relationships among all four dimensions of the food brand equity of a UNESCO Creative City of Gastronomy and tourist travel intentions. Consequently, this work proposes the following hypothesis:

**H6:**  *Brand equity of a UNESCO Creative City of Gastronomy positively influences tourist travel intentions.*

### 2.3. The Impact of Tourist Satisfaction on Travel Intentions

A review of previous research has shown that tourist satisfaction has a significant impact on increasing travel intentions [79]. Ravald and Grönroos identified tourist satisfaction as one of the most important factors in generating travel intentions, with satisfied tourists generating a greater degree of travel intentions than unsatisfied tourists [80]. Chi and Qu used Eureka Springs, Arkansas, USA as a case study to investigate the structural relationship between destination image, attribute satisfaction, overall satisfaction and tourist travel intentions, and found that destination image has an indirect effect on tourist travel intentions mainly through the mediating role of attribute satisfaction and overall satisfaction [81]. Tourist satisfaction can increase travel intentions towards a brand and generate repeat purchase intentions [79,82]. From a food tourism perspective, maximising tourists’ satisfaction with a UNESCO Creative City of Gastronomy is a key factor in ensuring travel intention. Therefore, the following hypothesis was proposed:

**H7:**  *Tourist satisfaction of a UNESCO Creative City of Gastronomy positively influences tourist travel intentions.*

**Figure 1:**
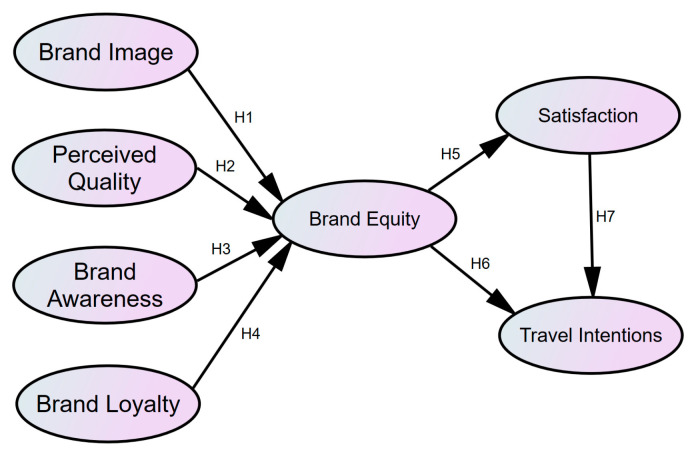
Theoretical model.

In summary, a research model of the relationship among brand equity, tourist satisfaction and travel intentions can be derived, as shown in Figure 1.

## 3. Methodology

This study employed a mixed-method sequential explanatory approach [83], using a quantitative research approach in the first phase with subsequent qualitative data used in the second phase to explain the quantitative results. The purpose of the initial quantitative phase was to validate the relationships between destination food brand equity, tourist satisfaction and travel intentions. The content of the semi-structured interviews was analysed during the qualitative phase to further clarify the relationships of the model. Using a combination of quantitative and qualitative methods allows for a more complete analysis of the research problem, where the weaknesses of one method are offset by the strengths of the other, and can provide more comprehensive knowledge for theory and practice [84].

### 3.1. Phase 1: Quantitative Research

In the initial stage, the empirical study used quantitative methods to test the conceptual model and its hypotheses. The quantitative procedures used in this study are described below.

#### 3.1.1. Measurements and Questionnaire Design

The questionnaire consisted of three main sections. The measurement scales in each section were drawn from previous studies for relevant research context and modified to fit the current study. The first section asked interviewees to indicate their perceptions of the four dimensions of the City of Gastronomy brand equity: brand image, perceived quality, brand awareness and brand loyalty. The second section asked interviewees to rate their satisfaction with the tourism they felt in the City of Gastronomy. The third section asked interviewees to express their travel intentions for that destination. Each variable measured a question using a seven-point Likert scale, ranging from completely disagree = 1 to completely agree = 7. The specific measurement items are shown in Table 1. The final section examines the demographics of the interviewees, including gender, age and education level.

#### 3.1.2. Data Collection

Before the formal research, 100 questionnaires were conducted for a pre-study in order to determine the reasonableness of the reserved question items. The formal research was conducted from March to April, 2022, and researchers invited tourists to fill in the questionnaire near famous restaurants in Yangzhou. Considering the potential impacts of the COVID-19 restrictions on tourist travel behaviours, the data for this study were collected over a longer period of time, covering eight weekends from March to April. To avoid subjectivity in the selection of interviewees, the study adopted the Brunt interval sampling method (Next-to-pass) [93], where the selected interviewees passed the screening questions. A total of 500 questionnaires were distributed and 481 valid questionnaires were returned, with a return rate of 96.20%.

#### 3.1.3. Data Analysis

To test the proposed hypotheses, the study used SmartPLS 3.0 software based on partial least squares to validate the structural equation model (SEM). Prior to testing the model, the samples were screened for missing values and outliers and subjected to a descriptive statistical analysis using SPSS 22.0 software. The reasons for using PLS-SEM rather than the widely used AMOS software in this study are 1. PLS-SEM is able to estimate the full range of path coefficients and multiple individual loadings simultaneously, thus allowing researchers to avoid biased and inconsistent parameter estimates [94]; 2. The PLS-SEM method does not require the data to obey a multivariate normal distribution and is able to handle different sample sizes; 3. In contrast to AMOS, which can only deal with reflective indicators, PLS-SEM is able to deal with two different types of indicators, formative and reflective [95]. As the brand equity in this study assumes that the brand equity in the model is a formative indicator, the PLS-SEM method is required.

### 3.2. Phase 2: Qualitative Research

In the second phase, this study used qualitative interviews to gain more insight into the quantitative findings from the first phase. Specifically, the aim of study 2 was to gain a deeper insight of why and how brand equity of the UNESCO Creative City of Gastronomy was connected to tourist satisfaction and travel intentions. Creswell argues that the analysis order enables researchers to gain a better understanding by examining different examples [96].

#### 3.2.1. Data Collection

After the completion of the quantitative questionnaire, the interviewees were invited to participate in semi-structured interviews so that the researcher could gain a deeper understanding of the tourists’ thoughts. Specifically, during the data collection of the questionnaire, each tourist was asked about their willingness to take part in further interviews. In total, 29 tourists expressed their willingness to participate in the interview. The entire interview process was recorded using a tape recorder. The questionnaire provided semi-structured questions that allowed interviewees to express their views openly (see Appendix A for details). This study also pre-studied the information gathered from the semi-structured interviews to assess the fluidity of the questions, refine the wording of the questions and eliminate any weaknesses in quality. The average duration of the interview was 25–30 min. According to the rule of theoretical saturation, interviews were stopped when no more new information emerged [97].

#### 3.2.2. Data Analysis

The audio files obtained from the interviews were transcribed and analysed using NVIVO 12.0. This study conducted the content analysis of the obtained text data after semi-structured interviews. A content analysis is a common method of observational research in a social science that applies to evaluate the symbolic content of all forms of recorded communication systematically [98]. A content analysis can provide qualitative explanations for the path relationships of structural equation models that make the researchers thoroughly understand the quantitative analysis of a Phase 1 [99].

## 4. Results

### 4.1. Profile of Samples

In terms of the composition of the sample (Table 2), the proportion of men and women is 48.44% and 51.56%, respectively, with little difference between the proportions of men and women. In terms of age, the majority are between 19 and 40 years old, of which 33.26% are between 19 and 25 years old, 30.15% are between 26 and 30 years old, 15.59% are between 31 and 40 years old and the rest account for 21.00%. In terms of education level, the sample was well educated, with 76.51% of interviewees having at least a university degree, which may be influenced by the expansion of higher education in China [100].

### 4.2. Common Method Bias

SPSS 22.0 was used for a common method bias test. Common method bias is a systematic error of artificial covariation between predictor and calibration variables that seriously confounds study results and potentially misleads conclusions. To ensure that this study is not affected, both procedural and statistical controls were employed. In terms of statistical control, this study used the Harman single-factor analysis in the data analysis, which was designed to ensure that the variance of the data was not explained by a single factor [101]. The results showed that the variance explained by a single factor was 29.076%, which was within the acceptable threshold (40%), meaning that a single factor did not account for most of the variance. Therefore, differences in commonly used methods are not considered in the data.

### 4.3. Measurement Model

A measurement model and structural model were used in the two-step approach to analyse the theoretical model presented in this study. The measuring model was assessed in order to confirm the formative and reflective constructs. The validity and reliability of constructs with reflecting measurement model requirements are shown in Table 3 below. Based on internal consistency reliability, indicator reliability, convergent validity and discriminant validity, the reflective construct’s reliability and validity were evaluated.

Firstly, as shown in Table 3, Cronbach’s alpha and composite reliability values of the constructs all exceeded 0.80, which met the criterion of greater than 0.7 [102], indicating that the scale had a good internal consistency. Secondly, the item loadings of each indicator are distributed between 0.812 and 0.871, which all meet the requirements of the indicator reliability test, manifesting that the reliability of each measurement item was high. Thirdly, the average variance extracted (AVE) of each dimension ranges from 0.674 to 0.724, all meeting the requirement of greater than 0.5 [103], demonstrating that the quantitative scale had a high convergent validity and the reliability of each latent variable was high. Lastly, based on Fornell and Larcker’s criterion, the correlation coefficients between all latent variables and other latent variables are less than the square root of each AVE, satisfying the requirements of convergent and discriminant validity tests (as shown in Table 4) [104]. Therefore, the reflective constructs of the model have a satisfactory discriminant validity.

In contrast to reflective indicators, formative indicators must avoid high levels of covariance because it will affect the relevance of weights [105]. The variance expansion factor (VIF) values for formative indicators ranged from 1.654 to 2.710, which is below the threshold of 5 [106], indicating that there was no potential co-linearity in the formative constructs studied. This study investigated the issue of co-linearity between latent variables. As a result, the evaluation can go on to the structural model since the assessments of the reflective and formative measuring models are both adequate.

### 4.4. Structural Model

**Figure 2:**
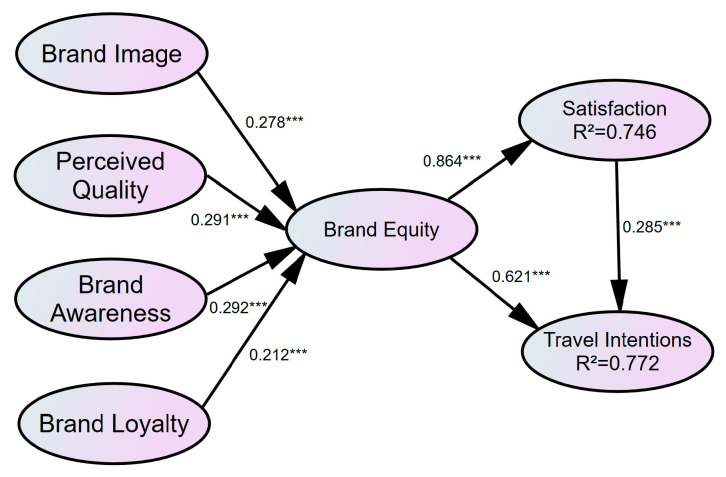
Results of the structural model test. Note: *** Significant at *p* < 0.01.

The endogenous latent variables were evaluated using the explainable variance (R2) as the basic evaluation criterion. Chin pointed out that when the explainable variance R2 value is greater than 0.33, it has a moderately strong explanatory power [106]. According to the calculation results of the bootstrapping method in this study, as shown in Table 5, the R2 values for the latent variables range from 0.746 to 0.772, all of which are greater than 0.33. Therefore, the structural model has a sufficient predictive validity. The Stone–Geisser’s Q2 value was calculated using the blindfolding technique to test the predictive relevance of the model [107]. When the Q2 value of a model is greater than 0, the predictive relevance of the change model is confirmed. Meanwhile, a bootstrap method based on 5000 resamples was used to test the significance level of the analysis. The t-values in the measurement model were distributed between 28.956 and 65.394, indicating a high level of significance of the observed variables. The t-values in the structural model were distributed between 3.992 and 61.476, with each path being more significant. The structural results of the proposed model are presented in Table 6 and Figure 2.

Specifically, brand image was shown to have a positive impact on brand equity, supporting Hypothesis 1 (β = 0.278, *p* < 0.01). Hypothesis 2 assumes the perceived quality has a significant impact on brand equity. The results supported Hypothesis 2 (β = 0.291, *p* < 0.01). Brand awareness positively influences brand equity (β = 0.292, *p* < 0.01), supporting Hypothesis 3. In addition, brand loyalty had a positive impact on brand equity (β = 0.212, *p* < 0.01), supporting Hypothesis 4. Hypotheses 5, 6 and 7 predicted the relationship between brand equity and tourist satisfaction as well as travel intentions. The results supported Hypothesis 5 at β = 0.864, *p* < 0.01; Hypothesis 6 at β = 0.621, *p* < 0.01; and Hypothesis 7 at β = 0.285, *p* < 0.01.

### 4.5. Qualitative Insights into the Quantitative Findings

The test results provide empirical data to support the hypothesised paths in this structural equation model. In order to further elucidate the internal mechanisms of the path relationships and better interpret the model fitting results, this study is complemented by a qualitative analysis of the content of semi-structured interviews to make up for the shortcomings of the quantitative study.

#### 4.5.1. Profile of Interviewees

Table 7 summarizes the profile information about the interviewees who participated in the qualitative study. In particular, the gender, age, education level and occupation are described in the table.

#### 4.5.2. The Impact of Food Brand Equity on Tourist Satisfaction

The quantitative analysis of the first phase showed that food brand equity of the UNESCO Creative City of Gastronomy shows a positive relationship with tourist satisfaction. When tourists perceive and appreciate the destination culinary brand equity, their satisfaction with the destination travel experience is likely to increase significantly. The four dimensions of food brand equity, based on the tourists’ perspective, are closely related to tourist satisfaction.

Food brand image consists of a variety of personal food perceptions that reflect or do not reflect objective reality [32]. The food brand image of a UNESCO Creative City of Gastronomy is assessed through physical factors, such as the food characteristics of the locals or influential events that occur in the destination [108]. Brand image has a positive impact on tourist satisfaction as it creates expectations prior to the visit and satisfaction depends on how this expectation compares to the actual visit experience [109]. The following two quotes confirm this finding:

*My impression of Yangzhou is that it is a quaint city in the south of the Yangtze River, with fine cuisine as well as rich and long-standing food culture. During my visit to Yangzhou, I was very satisfied with the taste of Yangzhou.*

(Interviewee 9, male, student)

*Yangzhou has been featured on many TV programmes and is now the UNESCO Creative City of Gastronomy. Therefore, visiting Yangzhou is a great opportunity to taste authentic Huaiyang cuisine. After a few days of tasting, it was really good and lived up to its reputation.*

(Interviewee 15, male, civil servant)

Perceived quality in this study refers to the ability of the UNESCO Creative City of Gastronomy to meet the expectations and needs of tourists to the cuisine. Perceived quality is a relatively stable perception of the tourism experience that affects the tourist’s experience of satisfaction or dissatisfaction over time [110]. Perceived quality is a prerequisite for satisfaction, and tourists’ satisfaction will increase if the destination meets their original expectations [111]. It is evidenced in two interviewees’ statements below:

*I went to one of the famous old restaurants in Yangzhou, which was bright and cleanly decorated with local characteristics, which caught my eye when I walked in. I am very conscious of the hygiene of my meals, so I pay extra attention to the environment of the restaurant. As it is an old restaurant, I was worried that the facilities might be too old before coming here, but now it seems I was worry too much.*

(Interviewee 5, male, freelancer)

*We came here to experience Yangzhou dim sum and the dining environment was very appetising and the staff were very attentive. In addition to that, the Yangzhou dim sum has some particularly good dishes such as the thousand-layer oil cake and the jade dumplings with reasonable price, which I was very pleased with.*

(Interviewee 19, male, employee)

Brand awareness reflects a tourist’s knowledge of a UNESCO Creative City of Gastronomy. The higher the brand awareness is, the more dominant the brand will be, which can further enhance tourists’ perceptions of satisfaction. The following statements from the interviewees are presented as evidence:

*Many people around me know that Yangzhou is the UNESCO Creative City of Gastronomy, and after posting the food here in my WeChat moments, many people have commented and interacted with me, which makes me feel very fulfilled and happy.*

(Interviewee 20, female, student)

*I came to Yangzhou for food. I really enjoy experiencing the culture represented by different cuisines. As the home of Huaiyang cuisine, Yangzhou fulfilled my requirement to experience authentic Huaiyang cuisine.*

(Interviewee 25, female, nurse)

*Yangzhou cuisine is so famous that there are even small restaurants in the city that specialize in selling Yangzhou fried rice. Many people, like me, must think of Yangzhou fried rice immediately when they think of fried rice. We were all very happy to have authentic Yangzhou fried rice on this trip.*

(Interviewee 2, male, employee)

Brand loyalty is a deep attachment between tourists and a UNESCO Creative City of Gastronomy, considered to be a reflection of tourists based on the brand experience [112]. Tourists with brand loyalty are more likely to trust a food brand when different options are available at the same time. Some tourists explained that having chosen a trusted food brand during their travel to a foreign place enhances the satisfaction that comes with the travel experience, making it easy to meet expectations and increase satisfaction. It is evidenced in two interviewees’ statements below:

*I once stayed in Yangzhou for half a month and the food I ate during that time is still unforgettable and very tasty. I didn’t hesitate to choose the local cuisine when I visited again after so many years, and many old restaurants had the same taste as that in those days. It was really satisfying to eat the food that I remembered from my heart, and all the good memories came back to me.*

(Interviewee 11, female, freelancer)

*Recognised by international organisations, Yangzhou cuisine has been featured on many TV programmes and has become a food capital known almost nationwide, so I am very confident about the future development of food tourism in Yangzhou. It was a very good decision to come here now to visit and taste the traditional food.*

(Interviewee 18, male, teacher)

#### 4.5.3. The Impact of Food Brand Equity on Tourist Travel Intentions

The previous discussion shows that food brand equity of the UNESCO Creative City of Gastronomy has a positive influence on tourists’ intentions to visit. The results of the data analysis indicated that four components of food brand equity were significant predictors of tourist travel intentions, namely brand image, perceived quality, brand awareness and brand loyalty, which is in line with previous researchers’ arguments [37,113].

Brand image, as the perception of a food brand in the minds of tourists [51], plays a crucial role in tourists’ evaluation of a UNESCO Creative City of Gastronomy, but is not the only dimension of food brand equity that tourists consider in the evaluation process [38]. In this study, when the food brand image of the UNESCO Creative City of Gastronomy becomes popular in the minds of tourists, it is naturally associated with a range of benefits, like the positive expectations about the destination’s cuisine, thereby increasing the intention to visit the UNESCO Creative City of Gastronomy [54]. For example, the following statements were made by two interviewees:

*I’ve been intrigued by the food here since I brushed up on Weibo that Yangzhou had become the UNESCO Creative City of Gastronomy. As I had time during this holiday, I brought my family along to Yangzhou.*

(Interviewee 14, male, freelancer)

*The internet says that Yangzhou dim sum is particularly good, so we came to sample these Yangzhou dim sum delicacies as recommended, like jadeite siu mai, big boiled shredded dried pork and crab yellow soup buns.*

(Interviewee 23, male, employee)

Perceived quality of food is the result of a visitor’s judgement comparing their expectations of the food experience with their perception of actual service performance [114]. The higher perceived quality of the food brand by tourists can increase their intention to recommend and revisit [60]. Some tourists explained that having a great dining experience while travelling makes them more likely to recommend the destination to others and they would like to come back to visit the destination themselves. One interviewee stated

*The food in Yangzhou is really delicious and true to its name, living up to its reputation as the food capital of the world. I read a lot of reviews before coming and they all recommended coming to Yangzhou for dim sum, so I was full of anticipation before coming. I enjoyed a lot of food on this trip and it was worth the trip. I will recommend my friends to come to Yangzhou.*

(Interviewee 27, male, student)

Brand awareness plays a key role in tourists’ intention to visit [115]. In this study, the higher the awareness is, the more dominant the food brand will be, which will influence tourists to add that food brand to the comparison of the same type of destination choices and ultimately influence the decision on travel intention [51], as evidenced by the following two interviewee quotes:

*When I think of Huaiyang cuisine, I think of the city of Yangzhou. We are from the north and usually rarely get to eat authentic Huaiyang cuisine. After Yangzhou became the UNESCO Creative City of Gastronomy, it became even more famous, and all my family and friends know about the city’s cuisine. In order to taste the authentic Yangzhou fried rice, crab roe soup buns and Yangzhou goose, we are ready to plan a trip here this holiday.*

(Interviewee 6, female, employee)

*Yangzhou is so famous for its “For River Town when willow-down and flowers reign”, an old poem that almost everyone knows. And, having filmed this place on the show “A Bite of China” that I watched earlier, it gave me a better understanding of Yangzhou’s cuisine and it would be a shame not to take a trip.*

(Interviewee 1, female, student)

Brand loyalty is a core component of brand equity [27]. From a food tourism perspective, food brand loyalty is the attitude of tourists who make the food brand of a UNESCO Creative City of Gastronomy their first choice or their willingness to pay a premium [116]. Tourists with high food brand loyalty will revisit or recommend the location to other potential tourists [70]. This is supported by the following statement:

*When I go back, I will recommend this place to my friends and relatives. The city of Yangzhou gives me a good feeling and is perfect for retirement because I think the food here is particularly health-conscious.*

(Interviewee 16, female, employee)

#### 4.5.4. The Impact of Tourist Satisfaction on Travel Intentions

The results of the quantitative data analysis showed that there is a positive relationship between tourist satisfaction with visiting a UNESCO Creative City of Gastronomy and travel intentions. Tourists who experience higher satisfaction during tourism are more likely to revisit the travel destination and spread positive word-of-mouth [116]. High satisfaction promotes an emotional bond and enhances connection with a food brand, leading to a high level of loyalty among tourists. It is evidenced in the following statements:

*During my 2 days in Yangzhou, I ate a lot of very famous food and was very satisfied and it was worth the trip. There were two restaurants that I was very impressed with. They were very special and I would go back if I had the chance to visit Yangzhou again.*

(Interviewee 28, male, freelancer)

*Coming to Yangzhou, the UNESCO Creative City of Gastronomy, eating and playing have left a very enjoyable holiday experience. I was able to try the Yangzhou dim sum that I had been longing for and it totally met my expectations before I came. I have rated it very highly on review sites and hope to introduce more people to Yangzhou cuisine.*

(Interviewee 13, female, doctor)

## 5. Discussion

This study sought to assess the role of brand equity in promoting food tourism intentions in the UNESCO Creative City of Gastronomy by testing the relationships between different dimensions of food brand equity, tourist satisfaction and travel intentions. Firstly, the findings suggest that all four dimensions of brand equity (brand image, perceived quality, brand awareness and brand loyalty) have a positive contribution to food brand equity, which is consistent with prior studies [19,27,35,38].

Secondly, as food brand equity increases, tourist satisfaction and travel intentions also increase. Local food in China makes a significant contribution to tourists’ travel satisfaction [117]. Horng et al.’s study confirmed that foreign tourists’ willingness to travel is based on their perception of Taiwan’s food brand equity, and that the brand equity of food ultimately affects tourists’ willingness to travel, which is consistent with the findings of this research [19]. Liu’s findings support the idea that a good brand equity and food appeal can more significantly influence foreign tourists’ behaviour [17]. Horng and Tsai argue that with the continuous development of food tourism, food plays an increasingly important role in increasing destination differentiation and enhancing tourists’ satisfaction and travel intention towards the destination [77]. Many destinations seek to use food as a key destination product to meet the growing food desires and interests of tourists [118]. Many aspects of food add great value to destinations, including economy, culture, destination identity and local sustainability [77]. Using mainland China as an example, Chen and Huang confirm the potential for destinations to use local food as a sustainable marketing tool [119]. The results of Apak and Gürbüz found that local food has a statistically significant and positive impact on sustainable tourism [3].

Thirdly, higher tourist satisfaction leads to higher travel intention. This result is similar to previous studies that have demonstrated the role of satisfaction in determining tourists’ future behavioural intentions [5,33,72,120]. All aspects of a destination have a positive impact on tourist satisfaction. Therefore, it is reasonable that food satisfaction, as a dimension of overall satisfaction, has a significant positive effect on tourists’ intention to visit. Meanwhile, Kivela and Crotts argue that tourists’ positive attitudes towards local cuisine may motivate them to revisit the destination and experience the cuisine [121]. Kim et al. noted that when tourists are satisfied with local dining, they are willing to show high levels of travel intentions [120]. Chen and Huang assessed the potential of local food as a destination attraction and noted the contribution of food to the development of local areas in China [117]. Therefore, in order to build a successful food brand, a UNESCO Creative City of Gastronomy should try to create a satisfying experience for tourists as satisfaction is an important factor in building a long-term relationship between tourists and the destination.

## 6. Conclusions and Implications

This study employed a structural model to assess the food brand equity of a destination from the tourists’ perspectives, and confirms its relationship with tourist satisfaction and travel intentions. With the utilization of a mixed research approach, this study focuses on examining the relationships between food brand equity, tourist satisfaction and travel intentions. Brand equity significantly and positively affects tourist satisfaction and travel intentions, and tourist satisfaction positively influences travel intentions. On this basis, corresponding strategies and suggestions are provided for Yangzhou to better develop its food tourism and build its food brand. The theoretical and practical implications of this study are as follows.

### 6.1. Theoretical Implications

The results of this study contribute to the existing literature. First, this study contributes to understanding the brand equity of the UNESCO Creative City of Gastronomy. From the available studies, scholars have expanded their research on the topic of brand equity to tourism, hospitality, sports and social networks [38,122,123,124]. However, few studies have analysed brand equity in the food domain. Up until now, no scholars have focused on the brand equity of a UNESCO Creative City of Gastronomy. This study expands the application of brand equity theory by analysing the dimensions of the brand equity of gastronomy based on tourists’ perspective, using Yangzhou, a UNESCO Creative City of Gastronomy, as a case study.

In addition, this study will help researchers and practitioners to better understand food brand equity based on empirical data. By providing a model to assess the relationship between brand equity dimensions, tourist satisfaction and travel intention, this study contributes to the tourism literature. By analysing the role of food brand equity dimensions in influencing tourist satisfaction and travel intention, this study provides a framework for researchers and related industry staff to visualize and understand the link between food brand equity, tourist satisfaction and travel intention.

Third, inspired by Truong et al.’s encouragement of mixed methods in tourism and hospitality, this study implemented a mixed-method sequential explanatory approach in Yangzhou, a UNESCO Creative City of Gastronomy [125]. This study supported the quantitative data with qualitative data and used semi-structured interviews to gain insight into tourists’ thoughts, providing an important addition to explain the pathways of action between model variables and a more comprehensive assessment of the theoretical model. This led to more rigorous and scientific conclusions of this study.

### 6.2. Practical Implications

This study can provide guidance for the development of a food brand in Yangzhou, and provide practical implications for other UNESCO Creative Cities of Gastronomy. Tourist satisfaction and travel intentions are based on tourists’ perceptions of food brand equity. Therefore, Yangzhou’s food brand equity will ultimately influence tourists’ travel intentions to visit Yangzhou. Keller argues that actively developing brand equity will yield better market benefits, and managers should focus on product and service brand differentiation [28]. The analysis provides recommendations for destination marketers on how to effectively use a food brand to retain and attract tourists.

First, destinations need focus on enhancing the food brand image. From this study, Yangzhou should make full use of its resources and cultural advantages to further develop its inherited famous dishes to create a distinctive food brand image to attract tourists [9,21]. The brand image constructed by the destination has a positive impact on the perceived value of the destination, which is an important component of brand equity [126]. By developing competitive specialties and services, Yangzhou can be associated with the image of Yangzhou cuisine as perceived and remembered by tourists, thus increasing its differentiation from other similar regions.

Second, it is significant to improve the perceived value of brand assets—not only the food itself but also the intangible services and tangible environment. Yangzhou should standardize the production process of cuisine, and improve the requirements for raw material intake so as to guarantee the quality and unique flavour of cuisine. In addition, service personnel in the catering industry ought to be trained regularly, improving their service awareness and level as well as realising the simultaneous enhancement of service specialisation and personalisation. At the same time, it is important to create an overall harmonious dining environment, construct themed restaurants with local cultural characteristics and focus on the protection of traditional food resources, especially long-established catering enterprises. The design style and internal furnishings of dining venues should be consistent with local food and cultural characteristics. Through some storytelling exhibitions about Yangzhou cuisine [127,128], tourists can feel the cultural connotations.

Third, efforts should be made to enhance brand awareness by continuously developing various activities related to the food brand. Unique branding activities can increase brand equity and create a competitive advantage in the tourism industry. Based on the positive relationship between an information search and food tourism engagement, destinations can use a range of different information access channels to distribute information [129]. A multi-channel approach should be used to promote Yangzhou cuisine, such as publishing brochures and magazines about Yangzhou’s food culture and speciality restaurants, shooting related documentaries or TV programmes and developing good relationships with travel agents [72]. It plays a significant role to work on positive word-of-mouth promotion of the Yangzhou cuisine to increase people’s willingness to the experience. It is also important to select tourists who have experienced Yangzhou cuisine for feedback, record the issues they mentioned, deal with them in a timely manner and monitor the evaluation to reduce negative word-of-mouth publicity about Yangzhou cuisine so as to improve its reputation among tourists.

Fourth, destinations should be committed to building brand loyalty among tourists and creating special experience programmes for the cuisine. The interactive experience of food can increase the participation and interactivity of tourists, making them subjectively more willing to get to know and experience Yangzhou cuisine in depth, increasing their satisfaction with the experience and thus their willingness to revisit the same place. At the same time, the existing food resources in Yangzhou are analysed and planned rationally to provide a unique experience for tourists by creating special food tourism routes. To change the single mode of dining in the past, modules of explanation, demonstration and interaction can be added to provide tourists with audio–visual enjoyment beyond tasting the food. This can increase their experience and knowledge of traditional culture, as well as create fond memories for tourists.

## 7. Limitations and Future Research

Despite the research contributions, several limitations of this study need to be considered to provide direction for future research. First, this study had a small age of interviewees during the quantitative phase, which may be overrepresented in the data set. More valid and reliable results could have been observed if the sample had been well distributed. Future studies should take care to balance the number of respondents in different age groups to avoid the resulting bias.

Second, this study was conducted in the UNESCO Creative City of Gastronomy of China, and there is concern that the results are not a better representative of a UNESCO Creative City of Gastronomy in other countries. Future studies could be replicated in other countries’ UNESCO Creative City of Gastronomy. In addition, the interviewees who participated in this study were limited to domestic Chinese tourists. The sample may not validate the broader generalizability of the results. The theoretical model in this study could be tested in the future with a different sample from different destinations or regions.

Finally, future research could extend the model so that additional factors play a mediating or moderating role in the relationship between brand equity, tourist satisfaction and travel intentions to elucidate other relationships not explored in the current paper.

## Figures and Tables

**Table 1:** The final items for the main survey.

Dimension	Items	References
Brand image	BI1: The actual experience of dining in Yangzhou is consistent with my impression of Yangzhou cuisine	Konecnik and Gartner [38], Horng et al. [19], Grace and O’ Cass [85]
	BI2: Yangzhou has a rich, diverse, unique and enticing cuisine	
	BI3: Yangzhou has a rich and long-standing food culture	
	BI4: A visit to Yangzhou is a great opportunity to sample the local cuisine	
Perceived quality	PQ1: Clean and tidy dining environment in Yangzhou	Boo et al. [37], Konecnik and Gartner [38], Horng et al. [19]
	PQ2: Quality restaurant services in Yangzhou	
	PQ3: Reasonably priced food tours in Yangzhou	
	PQ4: Yangzhou offers high-quality cuisine	
Brand awareness	BA1: Yangzhou cuisine enjoys an excellent reputation	Boo et al. [37], Yoo and Donthu [35], Oh [86], Bilgin [87], Horng et al. [19]
	BA2: Yangzhou cuisine enjoys a high reputation	
	BA3: I can quickly name the typical food of Yangzhou	
	BA4: When I think of cuisine, I immediately think of Yangzhou cuisine	
Brand loyalty	BL1: I would recommend others to come to Yangzhou for a food tour	Horng et al. [19], Arnett et al. [88], Boo et al. [37]
	BL2: I like to come to Yangzhou for food tours	
	BL3: I am very confident about the future of Yangzhou cuisine	
Tourist satisfaction	S1: The travel experience in Yangzhou brought me a high level of satisfaction	Baker and Crompton [89], Chen and Tsai [90]
	S2: A visit to Yangzhou is a joy	
	S3: It is a wise choice to visit Yangzhou	
	S4: My experience in Yangzhou met my expectations	
Travel intentions	TI1: I would recommend Yangzhou to others to visit and try Yangzhou food	Lam and Hsu [91], Yoo and Donthu [35], Žabkar et al. [92]
	TI2: I will actively promote visiting Yangzhou and tasting Yangzhou food	
	TI3: I hope to come back to Yangzhou to visit and taste the food	

**Table 2:** Demographic characteristics of sample (n = 481).

Demographic Characteristics		f	%
Gender	Male	233	48.44
	Female	248	51.56
Age	19–25	160	33.26
	26–30	145	30.15
	31–40	75	15.59
	41–50	46	9.57
	51–60	32	6.65
	More than 61	23	4.78
Education	Middle School and below	38	7.90
	High School	75	15.59
	Associate/Undergraduate Degree	345	71.73
	Postgraduate Degree and above	23	4.78

**Table 3:** Assessment of the reflective measurement model.

Dimensions	Items	Loading	Cronbach’s Alpha	Composite Reliability	Average Variance Extracted
Brand image	BI1	0.814	0.838	0.892	0.674
	BI2	0.833			
	BI3	0.812			
	BI4	0.824			
Perceived quality	PQ1	0.832	0.861	0.906	0.706
	PQ2	0.835			
	PQ3	0.835			
	PQ4	0.859			
Brand awareness	BA1	0.828	0.873	0.913	0.724
	BA2	0.851			
	BA3	0.871			
	BA4	0.854			
Brand loyalty	BL1	0.861	0.804	0.884	0.718
	BL2	0.847			
	BL3	0.834			

**Table 4:** Discriminant validity.

	BA	BI	BL	PQ
BA	0.851			
BI	0.808	0.821		
BL	0.774	0.815	0.847	
PQ	0.805	0.801	0.834	0.840

Note: The square root of AVE is shown in bold on the diagonal of the matrix.

**Table 5:** Coefficients of determination (R2) and prediction variance (Q2) of the construct.

Endogenous Latent Construct	Coefficients of Determination (R2)	Predictive Relevance (Q2)
Tourist satisfaction	0.746	0.547
Travel intentions	0.772	0.580

**Table 6:** Results of structural model and hypotheses’ testing.

Hypothesized Relationships	Coefficient	Results
H1: Brand image → Brand equity	0.278 ***	Accepted
H2: Perceived quality → Brand equity	0.291 ***	Accepted
H3: Brand awareness → Brand equity	0.292 ***	Accepted
H4: Brand loyalty → Brand equity	0.212 ***	Accepted
H5: Brand equity → Tourist satisfaction	0.864 ***	Accepted
H6: Brand equity → Travel intentions	0.621 ***	Accepted
H7: Tourist satisfaction → Travel intentions	0.285 ***	Accepted

Note: *** Significant at *p* < 0.01.

**Table 7:** Profile of qualitative study interviewees (n = 29).

Number	Gender	Age	Education	Profession
1	Female	21	Bachelor	Student
2	Male	28	Bachelor	Employees of private enterprises
3	Female	37	Postgraduate	Teacher
4	Female	19	Secondary studies	Student
5	Male	34	Bachelor	Freelancer
6	Female	46	Bachelor	Employees of private enterprises
7	Male	23	Bachelor	Student
8	Female	45	Secondary studies	Employees of private enterprises
9	Male	20	Bachelor	Student
10	Male	33	Postgraduate	Teacher
11	Female	50	Secondary studies	Freelancer
12	Female	23	Bachelor	Student
13	Female	42	Postgraduate	Doctor
14	Male	34	Bachelor	Freelancer
15	Male	47	Bachelor	Civil Servant
16	Female	38	Postgraduate	Employees of state enterprises
17	Female	45	Bachelor	Freelancer
18	Male	33	Postgraduate	Teacher
19	Male	27	Bachelor	Employees of state enterprises
20	Female	21	Bachelor	Student
21	Male	20	Bachelor	Student
22	Female	46	Postgraduate	Civil Servant
23	Male	32	Bachelor	Employees of private enterprises
24	Female	34	Bachelor	Freelancer
25	Female	26	Bachelor	Nurse
26	Female	47	Postgraduate	Doctor
27	Male	24	Bachelor	Student
28	Male	28	Bachelor	Freelancer
29	Female	34	Postgraduate	Teacher

## Data Availability

The data used to support the findings of this study can be made available by the corresponding author upon request.

## Appendix A

Core interview questions

We would like to seek your opinion on the following:

1. It is widely believed that the UNESCO Creative City of Gastronomy’s brand equity (e.g., brand image, perceived quality, brand awareness, brand loyalty) increases tourist satisfaction. Do you agree? If yes/no, why?
2. It is generally believed that the UNESCO Creative City of Gastronomy’s brand equity (e.g., brand image, perceived quality, brand awareness, brand loyalty) enhances tourist travel intentions. Do you agree? If yes/no, why?
3. It is widely believed that tourist satisfaction with the UNESCO Creative City of Gastronomy increases their travel intentions. Do you agree? If yes/no, why?
4. In terms of overall brand equity, it is widely accepted that there are four main components: brand image, perceived quality, brand awareness and brand loyalty. In your opinion, what is the most important component(s) that have the greatest impact on your satisfaction and travel intentions? (Can choose more than one if necessary)
5. With the impact of globalization and the influx of different kinds of cuisines into destinations, do you think UNESCO Creative Cities of Gastronomy can continue to maintain the uniqueness of its food brand equity?

## References

[1] Chang R.C., Mak A.H. (2018). Understanding gastronomic image from tourists’ perspective: A repertory grid approach. Tour. Manag..

[2] Savelli E., Gregory-Smith D., Murmura F., Pencarelli T. (2022). How to communicate typical-Local foods to improve food tourism attractiveness. Psychol. Mark..

[3] Apak Ö.C., Gürbüz A. (2023). The effect of local food consumption of domestic tourists on sustainable tourism. J. Retail. Consum. Serv..

[4] McKercher B., Okumus F., Okumus B. (2008). Food tourism as a viable market segment: It’s all how you cook the numbers!. J. Travel Tour. Mark..

[5] Choe J.Y.J., Kim S.S. (2018). Effects of tourists’ local food consumption value on attitude, food destination image, and behavioral intention. Int. J. Hosp. Manag..

[6] Şahin A., Kılıçlar A. (2023). The effect of tourists’ gastronomic experience on emotional and cognitive evaluation: An application of SOR paradigm. J. Hosp. Tour. Insights.

[7] Lai I.K.W. (2020). An examination of satisfaction on word of mouth regarding Portuguese foods in Macau: Applying the concept of integrated satisfaction. J. Hosp. Tour. Manag..

[8] Vesci M., Botti A. (2019). Festival quality, theory of planned behavior and revisiting intention: Evidence from local and small Italian culinary festivals. J. Hosp. Tour. Manag..

[9] Okumus F., Kock G., Scantlebury M.M.G., Okumus B. (2013). Using local cuisines when promoting small Caribbean Island destinations. J. Travel Tour. Mark..

[10] Piramanayagam S., Sud S., Seal P.P. (2020). Relationship between tourists’ local food experience scape, satisfaction and behavioural intention. Anatolia.

[11] Jeaheng Y., Han H. (2020). Thai Street food in fast growing global food tourism industry: Preference and behaviors of food tourists. J. Hosp. Tour. Manag..

[12] Seyitoğlu F., Ivanov S.H. (2020). A conceptual study of the strategic role of gastronomy in tourism destinations. Int. J. Gastron. Food Sci..

[13] Stalmirska A.M. (2020). Cultural globalisation and food in urban destination marketing. Tour. Geogr..

[14] Sürücü Ö., Öztürk Y., Okumus F., Bilgihan A. (2019). Brand awareness, image, physical quality and employee behavior as building blocks of customer-based brand equity: Consequences in the hotel context. J. Hosp. Tour. Manag..

[15] Jiang W.H., Li Y.Q., Liu C.H., Chang Y.P. (2017). Validating a multidimensional perspective of brand equity on motivation, expectation, and behavioural intention: A practical examination of culinary tourism. Asia Pac. J. Tour. Res..

[16] Liu C.H. (2020). Integration of different perspectives of culinary brand equity. J. Hosp. Tour. Manag..

[17] Liu C.H.S. (2016). The relationships among brand equity, culinary attraction, and foreign tourist satisfaction. J. Travel Tour. Mark..

[18] Stone M.J., Migacz S., Wolf E. (2019). Beyond the journey: The lasting impact of culinary tourism activities. Curr. Issues Tour..

[19] Horng J.S., Liu C.H., Chou H.Y., Tsai C.Y. (2012). Understanding the impact of culinary brand equity and destination familiarity on travel intentions. Tour. Manag..

[20] Bandarin F. (2012). Introduction: Series on urban creativity forum. City Cult. Soc..

[21] Chen Q., Huang R. (2016). Understanding the importance of food tourism to Chongqing, China. J. Vacat. Mark..

[22] Vuković A.J., Terzić A., Peštek A., Kukanja M., Renko S. (2020). Gastronomy and Regional Identity: Balkan versus National Cuisine. Gastronomy for Tourism Development.

[23] Hernandez-Rojas R.D., Folgado-Fernandez J.A., Palos-Sanchez P.R. (2021). Influence of the restaurant brand and gastronomy on tourist loyalty. A study in Cordoba’(Spain). Int. J. Gastron. Food Sci..

[24] Creative Cities Network: Gastronomy, UNESCO. https://en.unesco.org/creative-cities/creative-cities-map.

[25] Aulet S., Mundet L., Roca J. (2016). Between tradition and innovation: The case of El Celler De Can Roca. J. Gastron. Tour..

[26] Smith S.L.J., Xiao H. (2008). Culinary tourism supply chains: A preliminary examination. J. Travel Res..

[27] Aaker D.A. (1991). Managing Brand Equity.

[28] Keller K.L. (2003). Strategic Brand Management: Building, Measuring, and Managing Brand Equity.

[29] Kapferer J.N. (2008). The New Strategic Brand Management, Creating and Sustaining Brand Equity Long Term.

[30] Murphy L., Benckendorff P., Moscardo G. (2007). Destination brand personality: Visitor perceptions of a regional tourism destination. Tour. Anal..

[31] Oh H., Hsu C.H. (2014). Assessing equivalence of hotel brand equity measures in cross-cultural contexts. Int. J. Hosp. Manag..

[32] Aaker D.A. (1996). Building Strong Brands.

[33] Kim H.B., Kim W.G. (2005). The relationship between brand equity and firms’ performance in luxury hotels and chain restaurants. Tour. Manag..

[34] Bailey R., Ball S. (2006). An exploration of the meanings of hotel brand equity. Serv. Ind. J..

[35] Yoo B., Donthu N. (2001). Developing and validating a multidimensional consumer-based brand equity scale. J. Bus. Res..

[36] Camarero C., Garrido M.J., Vicente E. (2010). Components of art exhibition brand equity for internal and external visitors. Tour. Manag..

[37] Boo S., Busser J., Baloglu S. (2009). A model of customer-based brand equity and its application to multiple destinations. Tour. Manag..

[38] Konecnik M., Gartner W. (2007). Customer-based brand equity for a destination. Ann. Tour. Res..

[39] Faircloth J.B., Capella L.M., Alford B.L. (2001). The effect of brand attitude and brand image on brand equity. J. Mark. Theory Pract..

[40] Washburn J.H., Plank R.E. (2002). Measuring brand equity: An evaluation of a consumer-based brand equity scale. J. Mark. Theory Pract..

[41] Chang H.H., Liu Y.M. (2009). The impact of brand equity on brand preference and purchase intentions in the service industries. Serv. Ind. J..

[42] Williams P.W., Gill A.M., Chura N. (2004). Branding mountain destinations: The battle for placefulness. Tour. Rev..

[43] Analysis of the “Yangzhou ‘World Gourmet Capital’ Influence Survey” Report. http://www.yangzhou.gov.cn.

[44] King L.M., Halpenny E.A. (2014). Communicating the World Heritage brand: Visitor awareness of UNESCO’s World Heritage symbol and the implications for sites, stakeholders and sustainable management. J. Sustain. Tour..

[45] Poria Y., Reichel A., Cohen R. (2013). Tourists perceptions of World Heritage Site and its designation. Tour. Manag..

[46] Sassen (1991). The Global City: New York, London, Tokyo.

[47] Rosi M. (2014). Branding or sharing?. City Cult. Soc..

[48] Hankinson G. (2007). The management of destination brands: Five guiding principles based on recent developments in corporate branding theory. J. Brand Manag..

[49] Pearson D., Pearson T. (2017). Branding food culture: UNESCO creative cities of gastronomy. J. Food Prod. Mark..

[50] Yılmaz G., Kılıçarslan D., Caber M. (2020). How does a destination’s food image serve the common targets of the UNESCO creative cities network?. Int. J. Tour. Cities.

[51] Keller K.L. (1993). Conceptualizing, measuring, and managing customer-based brand equity. J. Mark..

[52] Lassar W., Mittal B., Sharma A. (1995). Measuring customer-based brand equity. J. Consum. Mark..

[53] Cai L.A. (2002). Cooperative branding for rural destinations. Ann. Tour. Res..

[54] Kotler N., Kotler P.H. (2001). Estrategiasy Marketing de Museos.

[55] Gartner W.C. (1994). Image formation process. J. Travel Tour. Mark..

[56] Lewis R.C., Chambers R.E. (1989). Marketing Leadership in Hospitality.

[57] Baalbaki S., Guzmán F. (2016). A consumer-perceived consumer brand-based brand equity scale. J. Brand Manag..

[58] Gartner W.C., Ruzzier M.K. (2011). Tourism destination brand equity dimensions: Renewal versus repeat market. J. Travel Res..

[59] Huang Y.H., Huang F.C. (2007). Tourists’ perceived value model and its measurement: An empirical study. Tour. Trib..

[60] Low G., Lamb C. (2000). The measurement and dimensionality of brand associations. J. Prod. Brand Manag..

[61] Cretu A.E., Brodie R.J. (2007). The influence of brand image and company reputation where manufacturers market to small firms: A customer value perspective. Ind. Mark. Manag..

[62] Lin Y.C. (2013). Evaluation of co-branded hotels in the Taiwanese market: The role of brand familiarity and brand fit. Int. J. Contemp. Hosp. Manag..

[63] Jago L., Chalip L., Brown G., Mules T., Ali S. (2003). Building events into destination branding: Insights from experts. Event Manag..

[64] San Martín H., Herrero A., García de los Salmones M.D.M. (2018). An integrative model of destination brand equity and tourist satisfaction. Curr. Issues Tour..

[65] Xu C.X., Mo L.P. (2014). A study about the driving factor model of tourism destination brand equity: A case study of Fenghuang. Tour. Trib..

[66] Buil I., Martinez E., de Chernatony L.D. (2013). The influence of brand equity on consumer responses. J. Consum. Mark..

[67] Harrington R.J., Ottenbacher M.C., Fauser S. (2017). QSR brand value: Marketing mix dimensions among McDonald’s, KFC, Burger King, Subway and Starbucks. Int. J. Contemp. Hosp. Manag..

[68] Flavian C., Martinez E., Polo Y. (2001). Loyalty to grocery stores in the Spanish market of the 1990s. J. Retail. Consum. Serv..

[69] Yuksel A., Yuksel F., Bilim Y. (2010). Destination attachment: Effects on customer satisfaction and cognitive, affective and conative loyalty. Tour. Manag..

[70] Yoon Y., Uysal M. (2005). An examination of the effects of motivation and satisfaction on destination loyalty: A structural model. Tour. Manag..

[71] Oliver R.L. (1980). A cognitive model of the antecedents & consequences of satisfaction decisions. J. Mark. Res..

[72] Assaf A.G., Josiassen A., Cvelbar L.K., Woo L. (2015). The effects of customer voice on hotel performance. Int. J. Hosp. Manag..

[73] Iglesias M.P., Guillen M.J.Y. (2004). Perceived quality and price: Their impact on the satisfaction of restaurant customers. Int. J. Contemp. Hosp. Manag..

[74] Keith J.E., Lee D., Lee R.G. (2004). The effect of relational exchange between the service provider and the customer on the customer’s perception of value. J. Relatsh. Mark..

[75] Seetharaman A., Nadzir Z.A.B.M., Gunalan S. (2001). A conceptual study on brand valuation. J. Prod. Brand Manag..

[76] Jang S., Namkung Y. (2009). Perceived quality, emotions, and behavioral intentions: Application of an extended Mehrabian-Russell model to restaurants. J. Bus. Res..

[77] Horng J.S., Tsai C.T. (2010). Government websites for promoting East Asian culinary tourism: A cross-national analysis. Tour. Manag..

[78] Murphy P., Pritchard M.P., Smith B. (2000). The distinction product & its impact on traveler perceptions. Tour. Manag..

[79] Thakur S., Singh A.P. (2012). Brand image, customer satisfaction and loyalty intention: A study in the context of cosmetic product among the people of central India. Int. J. Multidiscip. Manag. Stud..

[80] Ravald A., Grönroos C. (1996). The value concept and relationship marketing. Eur. J. Mark..

[81] Chi C.G.Q., Qu H.L. (2008). Examining the structural relationships of destination image, tourist satisfaction and destination loyalty: An integrated approach. Tour. Manag..

[82] Qiu H., Ye B.H., Bai B., Wang W.H. (2015). Do the roles of switching barriers on customer loyalty vary for different types of hotels?. Int. J. Hosp. Manag..

[83] Creswell J. (2005). Educational Research: Planning, Conducting, and Evaluating Quantitative and Qualitative Research.

[84] Hewlett D., Brown L. (2018). Planning for tranquil spaces in rural destinations through mixed methods research. Tour. Manag..

[85] Grace D., O’Cass A. (2005). Service branding: Consumer verdicts on service brands. J. Retail. Consum. Serv..

[86] Oh H. (2000). Diner’s perceptions of quality, value, & satisfaction. Cornell Hotel. Restaur. Adm. Q..

[87] Bilgin Y. (2018). The effect of social media marketing activities on brand awareness, brand image and brand loyalty. Business Manag. Studies Int. J..

[88] Arnett D.B., Laverie D.A., Meiers A. (2003). Developing parsimonious retailer equity indexes using partial least squares analysis: A method and applications. J. Retail..

[89] Baker D.A., Crompton J.L. (2000). Quality, satisfaction and behavioral intentions. Ann. Tour. Res..

[90] Chen C.F., Tsai D. (2007). How destination image and evaluative factors affect behavioral intentions?. Tour. Manag..

[91] Lam T., Hsu H.C. (2006). Predicting behavioral intention of choosing a travel destination. Tour. Manag..

[92] Žabkar V., Brenčič M.M., Dmitrović T. (2010). Modeling perceived quality, visitor satisfaction and behavioral intentions at the destination level. Tour. Manag..

[93] Brunt P. (1997). Market Research in Travel and Tourism.

[94] Loureiro S.M.C., Gonzalez F.J.M. (2008). The importance of quality, satisfaction, trust, and image in relation to rural tourist loyalty. J. Travel Tour. Mark..

[95] Hair J.F., Ringle C.M., Sarstedt M. (2011). PLS-SEM: Indeed a silver bullet. J. Mark. Theory Pract..

[96] Creswell J. (2014). Research Design: Qualitative, Quantitative, and Mixed Methods Approaches.

[97] Glaser B.G., Strauss A.L. (2017). Theoretical Sampling. Sociological Methods.

[98] Kolbe R.H., Burnett M.S. (1991). Content-analysis research: An examination of applications with directives for improving research reliability and objectivity. J. Consum. Res..

[99] Elo S., Kääriäinen M., Kanste O., Pölkki T., Utriainen K., Kyngäs H. (2014). Qualitative content analysis: A focus on trust worthiness. SAGE Open.

[100] Wang D., Liu D., Lai C. (2012). Expansion of higher education and the employment crisis: Policy innovations in China. Horizon.

[101] Podsakoff P.M., MacKenzie S.B., Lee J.Y., Podsakoff N.P. (2003). Common methodbias in behavioral research: A critical review of the literature and recommended remedies. J. Appl. Psychol..

[102] Nunnally J.C. (1978). Psychometric Theory.

[103] Henseler J., Ringle C.M., Sinkovics R.R. (2009). The use of partial least squares path modeling in international marketing. Adv. Int. Mark..

[104] Fornell C., Larcker D.F. (1981). Evaluating structural equation models with unobservable variables and measurement error. J. Mark. Res..

[105] Diamantopoulos A., Winklhofer H.M. (2001). Index construction with formative indicators: An alternative to scale development. J. Mark. Res..

[106] Chin W.W., Marcoulides G.A. (1998). The Partial Least Squares Approach to Structural Equation Modeling. Modern Methods for Business Research.

[107] Stone M. (1974). Cross-validatory choice and assessment of statistical predictions. J. R. Stat. Soc. B.

[108] Kashif M., Samsi S.Z.M., Sarifuddin S. (2015). Brand equity of Lahore Fort as a tourism destination brand. RAE-Rev. Adm. Empresas.

[109] Font X. (1997). Managing the tourist destination’s image. J. Vacat. Mark..

[110] Boulding William Ajay K., Richard S., Valarie A.Z. (1993). A Dynamic Process Model of Service Quality: From Expectations to Behavioral Intentions. J. Mark. Res..

[111] Wang T., Tran P.T.K., Tran V.T. (2017). Destination perceived quality, tourist satisfaction and word-of-mouth. Tour. Rev..

[112] Bloemer J., Ruyter K. (1998). On the relationship between store image, store satisfaction and store loyalty. Eur. J. Mark..

[113] Kandampully J., Juwaheer T.D., Hu H.H. (2011). The influence of a hotel firm’s quality of service and image and its effect on tourism customer loyalty. Int. J. Hosp. Tour. Adm..

[114] Bentzen E., Christiansen J.K., Varnes C.J. (2011). What attracts decision makers’ attention? Managerial allocation of time at product development portfolio meetings. Manag. Decis..

[115] Yuan J., Jang S. (2008). The effects of quality and satisfaction on awareness and behavioral intentions: Exploring the role of a wine festival. J. Travel Res..

[116] Zeithaml V.A., Berry L.L., Parasuraman A. (1996). The behavioural consequences of service quality. J. Mark..

[117] Chen Q., Huang R. (2018). Local food in China: A viable destination attraction. Br. Food J..

[118] Dinis I., Simoes O., Cruz C., Teodoro A. (2019). Understanding the impact of intentions in the adoption of local development practices by rural tourism hosts in Portugal. J. Rural. Stud..

[119] Chen Q., Huang R. (2019). Understanding the role of local food in sustaining Chinese destinations. Curr. Issues Tour..

[120] Kim S., Choe J.Y., Kim P.B. (2020). Effects of local food attributes on tourist dining satisfaction and future intention: The moderating role of food culture difference. J. China Tour. Res..

[121] Kivela J., Crotts J.C. (2006). Tourism and gastronomy: Gastronomy’s influence on how tourists experience a destination. J. Hosp. Tour. Res..

[122] Del Barrio G.S., Muñoz Leiva F., Golden L. (2020). A review of comparative advertising research 1975-2018: Thematic and citation analyses. J. Bus. Res..

[123] Wang M.C.H., Tang Y.Y. (2018). Examining the antecedents of sport team brand equity: A dual-identification perspective. Sport Manag. Rev..

[124] Dwivedi A., Johnson L.W., Wilkie D.C., De Araujo-Gil L. (2019). Consumer emotional brand attachment with social media brands and social media brand equity. Eur. J. Mark..

[125] Truong D., Liu R.X., Yu J.J. (2020). Mixed methods research in tourism and hospitality journals. Int. J. Contemp. Hosp. Manag..

[126] Jara M., Cliquet G. (2012). Retail brand equity: Conceptualisation and measurement. J. Retail. Consum. Serv..

[127] Aguilera A.P. (2018). Food Stories: A Design Method for Understanding Meaning through Identity, Emotion, and Experience. Ph.D. Thesis.

[128] Park E., Muangasame K., Kim S. (2021). ‘We and our stories’: Constructing food experiences in a UNESCO gastronomy city. Tour. Geogr..

[129] Karim S.A., Chi C.G.Q. (2010). Culinary tourism as destination attraction: An empirical examination of destinations’ food image. J. Hosp. Mark. Manag..

